# Image Encryption Scheme Based on Mixed Chaotic Bernoulli Measurement Matrix Block Compressive Sensing

**DOI:** 10.3390/e24020273

**Published:** 2022-02-14

**Authors:** Chen Yang, Ping Pan, Qun Ding

**Affiliations:** Electronic Engineering College, Heilongjiang University, Harbin 150080, China; 2201658@s.hlju.edu.cn (C.Y.); 2201647@s.hlju.edu.cn (P.P.)

**Keywords:** image encryption, block compressive sensing, Bernoulli measurement matrix, chaotic map, two-way diffusion

## Abstract

Many image encryption schemes based on compressive sensing have poor reconstructed image quality when the compression ratio is low, as well as difficulty in hardware implementation. To address these problems, we propose an image encryption algorithm based on the mixed chaotic Bernoulli measurement matrix block compressive sensing. A new chaotic measurement matrix was designed using the Chebyshev map and logistic map; the image was compressed in blocks to obtain the measurement values. Still, using the Chebyshev map and logistic map to generate encrypted sequences, the measurement values were encrypted by no repetitive scrambling as well as a two-way diffusion algorithm based on GF(257) for the measurement value matrix. The security of the encryption system was further improved by generating the Secure Hash Algorithm-256 of the original image to calculate the initial values of the chaotic mappings for the encryption process. The scheme uses two one-dimensional maps and is easier to implement in hardware. Simulation and performance analysis showed that the proposed image compression–encryption scheme can improve the peak signal-to-noise ratio of the reconstructed image with a low compression ratio and has good encryption against various attacks.

## 1. Introduction

With the rapid development of the internet and computer technology, more images, videos, and other multimedia information are being transmitted and stored through the internet. Image information security is receiving more attention. Image encryption is an effective method to protect digital images [1,2,3,4,5,6].

Image encryption methods are currently available using various techniques, such as chaotic systems [7,8,9,10,11,12,13,14,15,16,17], DNA coding [18,19,20,21], substitution boxes [22,23,24], and cellular automata [25,26,27,28]. Chaotic phenomena [29,30] are seemingly random irregular motions that occur in a deterministic system—a system described by a deterministic theory that behaves in an uncertain, irreducible, and unpredictable manner. Chaotic systems are the most widely used and continuously improved systems in the field of image encryption. Chaotic systems for image encryption are classified into two categories: one-dimensional (1D) chaotic systems [7,8,9,10,11] and multidimensional (MD) chaotic systems. One-dimensional chaotic systems have advantages, such as simple structures, easy implementation, and low computational complexity [11], but they also have disadvantages, such as small key spaces and low security [8,9]. To solve this problem, some MD chaotic systems have been applied to image encryption [12,13,14,15,16,17]. The encryption algorithms based on MD systems have larger key spaces and higher security levels; however, MD chaotic systems are relatively difficult to implement in hardware or software and the respective encryption systems have high computational complexity [11]. In this paper, we used Chebyshev mapping and logistic mapping to generate chaotic sequences for the encryption process and calculate the initial value of the one-dimensional chaotic system by the Secure Hash Algorithm-256(SHA-256) function, which is not only easy to implement and increase the key space, but it also reduces the computational complexity of the cryptosystem and improves the security of the algorithm.

As a revolutionary data acquisition technique, compressive sensing (CS) [31,32,33] opens new perspectives for simultaneous compression and encryption of plaintexts, where the measurement matrix is shared between the sender and receiver as a key. To improve the confidentiality, linear feedback shift registers, pseudo-random number generators, and chaotic mappings were proposed to construct the measurement matrix [34,35,36,37,38]. The design of the measurement matrix is the key to ensuring the quality of signal sampling and to determine the hardware implementation of compressed sampling.

The methods used for constructing the measurement matrix are divided into two categories. One is the random measurement matrix, which mainly includes Gaussian measurement matrix, Bernoulli measurement matrix, local Fourier measurement matrix, etc. [32,39]. Although these matrices can reconstruct the original signals well, they are difficult to implement in hardware, in practical applications; storing the matrix elements requires a large storage space. Another type of matrix is the deterministic measurement matrix. In constructing such a matrix, once the system parameters and the parameters of the construction are determined, the elements of the matrix are also determined. The chaotic sequence generated by the chaotic system is a unification of determinism and randomness, and the measurement matrix constructed from the chaotic sequence is a deterministic matrix, which can effectively solve the problems of instability and the need for multiple measurements in the common random measurement matrix in experiments. Therefore, some scholars proposed to use chaotic sequences for the construction of compressive–aware measurement matrices [40,41,42,43,44]. L. Yu proposed using logistic chaotic sequences to construct measurement matrices in the literature [40] and proved that the matrices satisfied the RIP condition; Frunzete [41] et al. proved that the measurement matrices constructed by using tent chaotic sequences can satisfy the RIP condition. However, all of the above measurement matrix construction methods use chaotic sequences generated by a single chaotic mapping to construct the measurement matrix. The performance of the chaotic sequence generated by a single chaotic mapping is slightly worse than that of the hybrid chaotic sequence, so the performance of the measurement matrix is again influenced by the performance of the chaotic sequence [43]. In order to avoid affecting the reconstruction effect of the signal due to the lack of performance of a single chaotic sequence, a measurement matrix construction algorithm based on the XOR operation chaotic sequence is proposed to improve the reconstruction performance of the measurement matrix and the easy implementation feature of the hardware.

To reduce the computational complexity and storage space, the block compressed sensing (BCS) framework was introduced to process medium to large images. R. Huang et al. [45] designed a block cipher structure consisting of linear measurement, scrambling, S-box, and XOR operations. This scheme can work efficiently in the parallel computing environment. Zhu L et al. [46] conducted a novel BCS-based encryption scheme. The nonuniform sampling strategy was adopted in BCS stage to improve the compression efficiency, and the permutation–diffusion framework was utilized to enhance security. Experiments show that the algorithm has good reconstruction capability, but it has low correlation with plaintext images, and is vulnerable to known plaintext and selected plaintext attacks.

In this paper, we propose a simple and secure image encryption algorithm based on BCS and one-dimensional chaotic mapping. This scheme is made up of two primary stages. In the first stage, after generating chaotic sequences using two simple one-dimensional chaotic mappings—a Chebyshev map and logistic map for binary quantization and XOR operations—a Bernoulli measurement matrix was generated and the measurement value matrix was obtained by performing BCS on the original image. In the second stage, still using the Chebyshev map and logistic map to generate chaotic sequences, the measurement value matrix was encrypted to obtain the ciphertext image. The simulation results verify the security, effectiveness, and good reconfiguration performance of the scheme. The main contributions of this paper are as follows.

1. A hybrid chaotic sequence measurement matrix satisfying Bernoulli’s property based on Chebyshev map and a logistic map, which is easy to implement in hardware, is designed, and the performance of the resulting measurement matrix is analyzed and applied to BCS.
2. The SHA-256 value of the plaintext image is used to calculate the initial value of the chaotic mapping for the encryption algorithm part, which increases the relevance of the algorithm to the plaintext image and improves the ability of the scheme to resist the selective plaintext attack.
3. A “no repetition” scrambling algorithm and a Galois domain-based two-way diffusion algorithm are proposed to encrypt the measurement value matrix and improve the security of the BCS framework.
4. In the reconstruction stage, the SPL reconstruction algorithm, based on DDWT, is used, which, combined with the hybrid chaotic sequence measurement matrix proposed in this paper, can achieve a much higher reconstruction quality of decrypted images with low compression ratio.

The rest of this paper is organized as follows. The preliminaries are discussed in Section 2. The proposed algorithm is described in Section 3. Simulation results and discussion are given in Section 4, and the statistical analysis of the scheme is discussed in Section 5. Conclusions are given in Section 6.

## 2. Preliminaries

In this section, BCS and the measurement matrix generation algorithm will be briefly introduced.

### 2.1. Block Compressive Sensing

In traditional compressive sensing (CS) image reconstruction methods, the observations are obtained by randomly projecting the image as a whole through the observation matrix. However, two-dimensional image signals often contain a large amount of information, which make the overall projection of the data very large, requiring large storage space and leading to high computational complexity in the image reconstruction process, causing a lot of problems for the specific implementation. Therefore, the compressed perception theory usually encounters two major obstacles when applied to video and images [47]: first, the observation matrix occupies a large amount of storage; second, the computational complexity is too high in the image reconstruction process. To solve this problem, the block compressive sensing (BCS) framework [48] is proposed, which is described in detail as follows.

The image x, with pixel $N=I_{r}\times I_{c}$, is divided into several B×B size image blocks. Denoting the *i*-th image block by $x_{i}$, $i=1,2,\cdots ,N/B^{2}$. The same measurement matrix $\Phi_{B}$ is applied to each sub-block $x_{i}$ for the chunked image to obtain the measurement values:

(1) $$y_{i}=\Phi_{B}x_{i}$$

where $\Phi_{B}\in R^{M_{B}\times B^{2}}$, and $M_{B}=\left\lfloor CR\times B^{2}/N\right\rfloor$ is the number of samples of each sub-block and *CR* is the compression ratio. The image is chunked; equivalently, the sampling matrix of the entire image is:

(2) $$\Phi =\left[\begin{array}{cccc} \Phi_{B} & & & \\ & \Phi_{B} & & \\ & & \ddots & \\ & & & \Phi_{B} \end{array}\right]$$

The observed values y for the whole image are:

(3) $$y=\Phi x=\left[\begin{array}{c} \Phi_{B}x\\ \vdots \\ \Phi_{B}x \end{array}\right]$$

Compared to the traditional image compressive sensing algorithm, the BCS algorithm solves the problem of large memory consumption required for the observation matrix in the image reconstruction process and greatly improves the speed of the image compressive sensing signal recovery. At the same time, the receiver does not have to wait for all of the sampling data transmissions to be completed, which is convenient for real-time processing of the signal.

Reconstruction algorithms for block compressive sensing can use orthogonal matching pursuit (OMP) [49], subspace pursuit (SP) [50], basis pursuit (BP) [51], etc., and can effectively recover the signal from the measured values.

In this paper, we use a smoothed PL (SPL) based on a dual-tree discrete wavelet transform (DDWT) [52] to reconstruct the image. A block-based random image sampling was coupled with a projection-driven compressed-sensing recovery that encouraged sparsity in the domain of directional transforms simultaneously with a smooth reconstructed image. Both contourlets, as well as complex-valued dual-tree wavelets, were considered for their highly directional representations, while bivariate shrinkage was adapted to their multiscale decomposition structures to provide the requisite sparsity constraint. Smoothing was achieved via a Wiener filter incorporated into the iterative projected Landweber compressed-sensing recovery, yielding fast reconstruction. The proposed approach yields images with qualities that match or exceed that produced by a popular, yet computationally expensive, technique, which minimizes total variation. Additionally, reconstruction quality is substantially superior to that from several prominent pursuits-based algorithms that do not include any smoothing.

### 2.2. Measurement Matrix Generation Algorithm

#### 2.2.1. Hybrid Chebyshev–Logistic Bernoulli Sequence

The Chebyshev chaotic map is defined as:

(4) $$x_{n+1}=\cos\left(\omega \cdot \arccos x_{n}\right)\;x_{n}\in \left[-1,1\right]$$

where ω is the order of the system. When the order ω>2, the map enters the chaotic state and the chaotic sequence can be generated by Equation (4). When ω>4, the Chebyshev map is in the full-rank mapping state, and the chaotic sequence generated by the system iteration is traversed on the interval [−1, 1]. The mean value of the chaotic sequence generated by the Chebyshev map is 0, and its probability density function is:

(5) $$\rho_{X}\left(x\right)=\left\{ \begin{array}{ll} \frac{1}{\pi \sqrt{1-x^{2}}}, & x\in (-1,1)\\ 0, & \mathrm{else} \end{array}\right.$$

The logistic chaotic map is defined as:

(6) $$y_{n+1}=1-\lambda y_{n}^{2},y_{n}\in \left[-1,1\right]$$

where λ is the system parameters, when λ∈(1.40015,2], the Lyapunov exponent of the system are greater than 0, then the mapping is in the chaotic state. When λ=2, the logistic map is a full mapping, the chaotic sequence generated by the iteration of Equation (6) has good pseudo-randomness, and the generated chaotic sequences are distributed in the interval [−1, 1]. The probability density function of the logistic chaotic sequence is:

(7) $$\rho_{X}\left(y\right)=\left\{ \begin{array}{ll} \frac{1}{\pi \sqrt{1-y^{2}}}, & y\in (-1,1)\\ 0, & \mathrm{else} \end{array}\right.$$

The chaotic sequence generated by the above chaotic system is a real-valued sequence, which is not suitable for transmission and processing in digital communication systems, so it is necessary to convert the real-valued chaotic sequence into a binary chaotic sequence. Here, the signum can be used to convert the chaotic sequence into a binary chaotic sequence. The signum formula is shown in Equation (8).

(8) $$\mathrm{sgn}(x)=\{\begin{array}{ll} 0 & x<c\\ 1 & x\geq c \end{array}$$

where *c* is the threshold value and *x* is the value of the real-valued sequence.

**Theorem** **1.** *The chaotic sequence*$\left\{x_{n}\right\}$*generated by the Chebyshev chaotic map, is transformed by the signum function to produce a spread spectrum sequence*$\left\{x_{n}^{\prime }\right\}$*satisfying the Bernoulli distribution, which is called the Chebyshev*–*Bernoulli (C*–*B) sequence.*

**Theorem** **2.** *The chaotic sequence*$\left\{y_{n}\right\}$*generated by the iteration of the logistic chaotic map, is transformed by the signum function to generate the spread spectrum sequence*$\left\{y_{n}^{\prime }\right\}$, *which is a Bernoulli random sequence; that is, logistic*–*Bernoulli (L*–*B) sequence.*

**Theorem** **3.** *The sequence generated by XOR operation of two Bernoulli random sequences is still the Bernoulli random sequence.*

Equation (5) shows that the Chebyshev chaotic sequence $\left\{x_{n}\right\}$ is uniformly distributed in [−1, 1]. From Equations (5) and (8), the distribution of $x_{n}$ on the interval [−1, 1] corresponding to a value of 0 or 1 for $x_{n+1}^{\prime }$ can be found as follows:

(9) $$\mathrm{sgn}\left(x_{n+1}^{\prime }\right)=\{\begin{array}{ccc} 0, & & x_{n}\in \left(-1,0\right)\\ 1, & & x_{n}\in \left(0,1\right) \end{array}$$

Similarly, the logistic chaotic sequence $\left\{y_{n}\right\}$ is uniformly distributed on [−1, 1]. The distribution of $y_{n}$ on [−1, 1] corresponding to $y_{n+1}^{\prime }$ when it is 0 and 1, respectively, can be derived from Equations (12) and (13).

(10) $$\mathrm{sgn}\left(y_{n+1}^{\prime }\right)=\{\begin{array}{ccc} 0, & & y_{n}\in \left(-1,-\frac{\sqrt{2}}{2}\right)\cup \left(\frac{\sqrt{2}}{2},1\right)\\ 1, & & y_{n}\in \left(-\frac{\sqrt{2}}{2},\frac{\sqrt{2}}{2}\right) \end{array}$$

Finally, a hybrid chaotic spread spectrum sequence is generated, as shown in Equation (11).

(11) $$z_{n}=x_{n}^{\prime }⊕y_{n}^{\prime }$$

XOR operation does not change the state of the sequence distribution. Therefore, the sequence $\left\{z_{n}\right\}$ is also uniformly distributed over [−1, 1]. From Equations (9) and (10), the distribution of $z_{n}$ on [−1, 1] corresponding to $z_{n+1}$ taking values of 0 and 1 can be obtained as:

(12) $$\mathrm{sgn}\left(z_{n+1}\right)=\{\begin{array}{lll} 0, & & z_{n}\in \left(-1,-\frac{\sqrt{2}}{2}\right)\cup \left(0,\frac{\sqrt{2}}{2}\right)\\ 1, & & z_{n}\in \left(-\frac{\sqrt{2}}{2},0\right)\cup \left(\frac{\sqrt{2}}{2},1\right) \end{array}$$

From Equation (12), the probability that $z_{n+1}$ takes the value of 0 or 1 is 1/2. Treating $\left\{z_{n}\right\}$ as a Markov chain, its one-step transfer probability matrix is:

(13) $$P=\left[\begin{array}{cc} 0.5 & 0.5\\ 0.5 & 0.5 \end{array}\right]$$

The i-step transfer probability matrix $P^{i}=P\left(i\geq 1\right)$ can be obtained from Equation (13). According to the properties of the Markov chain, it can be proved that the sequence $\left\{z_{n}\right\}$ is random and independent statistics, so the sequence $\left\{z_{n}\right\}$ is the Bernoulli sequence, and it is called the mixed Chebyshev and logistic Bernoulli (MCLB) sequence.

#### 2.2.2. Construction of a Measurement Matrix Based on MCLB Sequence

**Figure 1:**
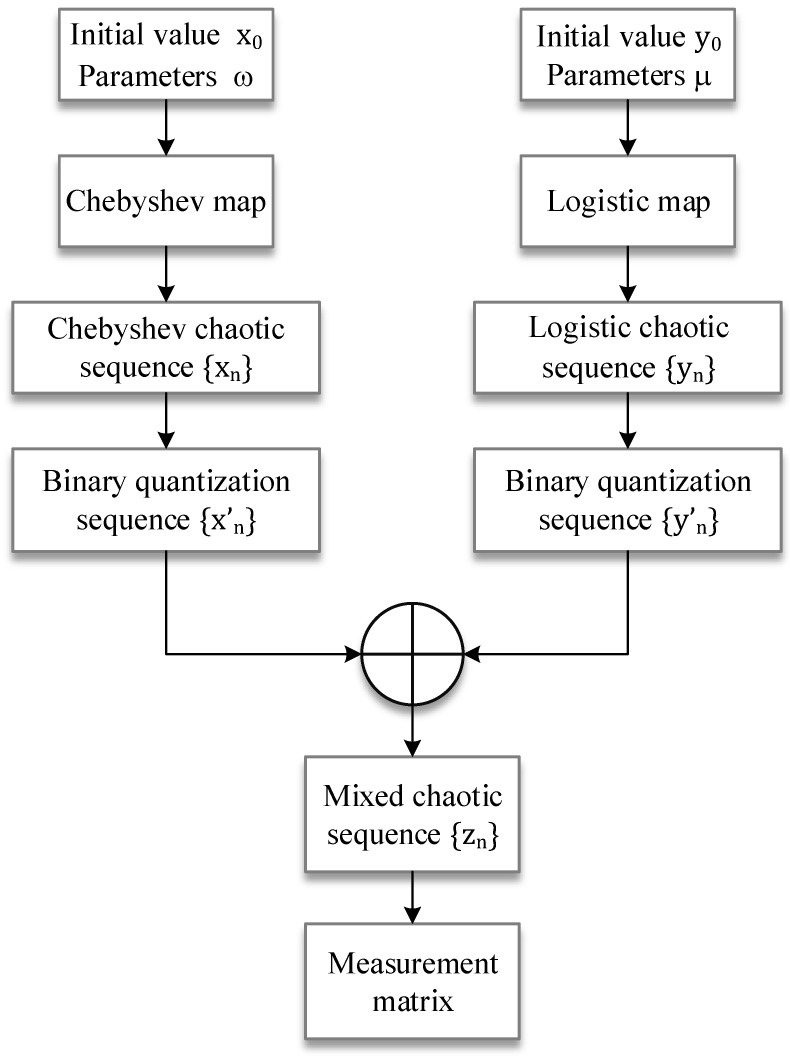
Flowchart of the constructing the measurement matrix based on MCLB sequences.

The Bernoulli random observation matrix, as a common random measurement matrix, can satisfy the RIP property with great probability [53]. The mixed chaotic sequence proposed in the previous subsection is a random and statistically independent Bernoulli random sequence, so the measurement matrix constructed from the hybrid chaotic sequence satisfies the RIP property. Therefore, this paper proposes an algorithm to construct the measurement matrix based on the mixed chaotic sequence, and its flowchart is shown in Figure 1.

The specific construction steps are as follows:

1. Set the parameters ω=4, λ=2 of Equations (4) and (6), and given the initial values $x_{0}$, $y_{0}$, generate Chebyshev chaotic sequences $\left\{x_{n}\right\}$ and logistic chaotic sequences $\left\{y_{n}\right\}$ of the desired length iteratively from Equations (4) and (6).
2. Binary quantization of the chaotic sequences $\left\{x_{n}\right\}$ and $\left\{y_{n}\right\}$ using Equations (9) and (10), respectively, to obtain the chaotic spread spectrum sequence $\left\{x_{n}^{\prime }\right\}$ and $\left\{y_{n}^{\prime }\right\}$, and XOR operation to generate a hybrid chaotic sequence $\left\{z_{n}\right\}$.
3. The mixed chaotic sequence is cyclically shifted by columns to generate a measurement matrix of desired size, which is called the mixed Chebyshev and logistic Bernoulli matrix (MCLBM). Finally, the MCLBM is orthogonalized and normalized to obtain the final measurement matrix.

#### 2.2.3. Simulation Test Based on MCLBM

This section verifies the reliability and validity of the MCLBM through image simulation experiments and compares the MCLBM with the Gaussian matrix (GM), Bernoulli matrix (BM), logistic–Bernoulli matrix (LBM), and Chebyshev–Bernoulli matrix (CBM) are compared.

BCS was used to test the “Lena” image with a size of 512×512. First, the image was divided into 32×32 blocks, the compression ratio was set to 0.3, different measurement matrices were used for compression measurements, and the DDWT-SPL algorithm was used in the reconstruction stage. MCLBM, GM, BM, LBM, and CBM were selected as measurement matrices for compressive reconstruction of “Lena” to compare the performance. In order to reduce the instability of GM and BM, they were averaged over 100 experiments. Given the parameters of the chaotic system, the constructed measurement matrices were intrinsically deterministic and were definite measurement matrices, so there was no need to repeat the experiments. We used the peak signal-to-noise ratio (PSNR) as an evaluation index for reconstructed images. The PSNR was the most widely used objective evaluation index for images. The PSNR is usually used to judge the quality of the decrypted image [54]. The larger the PSNR value, the smaller the difference between the two images and the higher the reconstruction accuracy.

PSNR is defined as follows:

(14) $$\mathrm{PSNR}=10\log\frac{255\times 255}{\left(\frac{1}{M\times N}\right)\sum_{i=1}^{M}\sum_{j=1}^{N}{\left(X\left(i,j\right)-Y\left(i,j\right)\right)}^{2}}$$

where X(i,j) is the value of the corresponding pixel point in the original image and Y(i,j) is the value of the corresponding pixel point in the reconstructed image.

**Figure 2:**
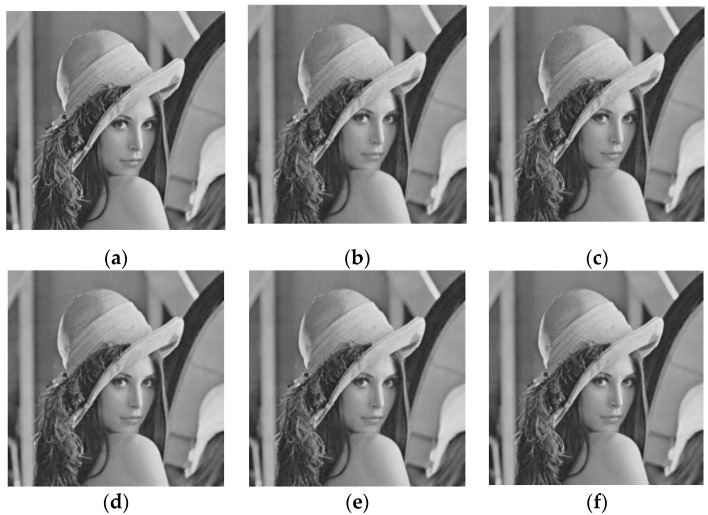
Reconstructed images with different measurement matrices at a compression ratio of 0.3. (**a**) Original image; (**b**) GM reconstructed image; (**c**) BM reconstructed image; (**d**) LBM reconstructed image; (**e**) CBM reconstructed image; (**f**) MCLBM reconstructed image.

Figure 2 shows the effect plots of the reconstruction of the “Lena” image with different measurement matrices for a compression ratio of 0.3 compared with the original image. It can be seen from the figure that MCLBM has the best reconstruction effect and is closer to the original image.

For a more intuitive understanding of the performance of MCLBM, the experimental data of PSNR and reconstruction time of the five measurement matrices in reconstructing images at a compression ratio of 0.3 are given in Table 1. From the data in the table, it can be obtained that MCLBM outperforms several other measurement matrices in terms of PSNR at a compression ratio of 0.3. The reconstruction times of different measurement matrices are approximately the same, and the reconstruction time of MCLBM is slightly better than the other measurement matrices.

**Figure 3:**
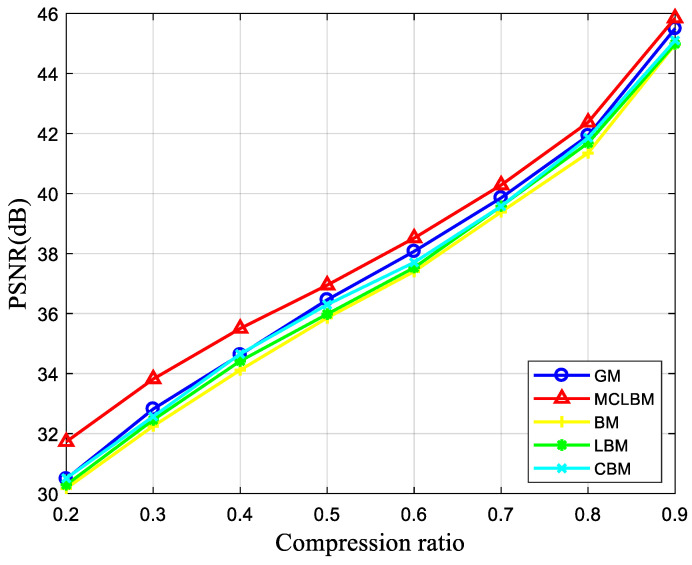
PSNR using different measurement matrices at different compression ratios.

Figure 3 reflects the variation of the PSNR of the “Lena” reconstructed images for different compression ratios for the five measurement matrices GM, BM, LBM, CBM, and MCLBM. As can be seen in Figure 3, the higher the compression ratio, the higher the PSNR of the reconstructed image when using different measurement matrices to compress the reconstructed image. Before the compression ratio is less than 0.4, the PSNR of the reconstructed image with MCLBM as the measurement matrix is greater than that of other measurement matrices by about 1 dB. When the compression ratio is greater than 0.4, the effect of the compression ratio on the PSNR exceeds that of the measurement matrix, so the PSNR with MCLBM as the measurement matrix is not greater than that of other measurement matrices by 1 dB, but is still greater than that of other measurement matrices. In summary, the peak signal-to-noise ratio of the reconstructed image using MCLBM is always the largest, and the reconstruction effect is better than the other four measurement matrices.

## 3. The Proposed Algorithm

The main objective of the image compression/encryption algorithm proposed in this paper was to improve the performance of image compression, reconstruction, and encryption at low compression ratios; that is, with a reduced amount of more data. For this purpose, the plaintext images were blocked and measured using the MCLBM in the compressive measurement phase. To improve the resistance to selective plaintext attacks, the chaotic encryption sequence was generated for the plaintext image using the SHA-256 function to generate a key as the initial value of the chaotic map, and a new image encryption scheme was designed accordingly.

**Figure 4:**
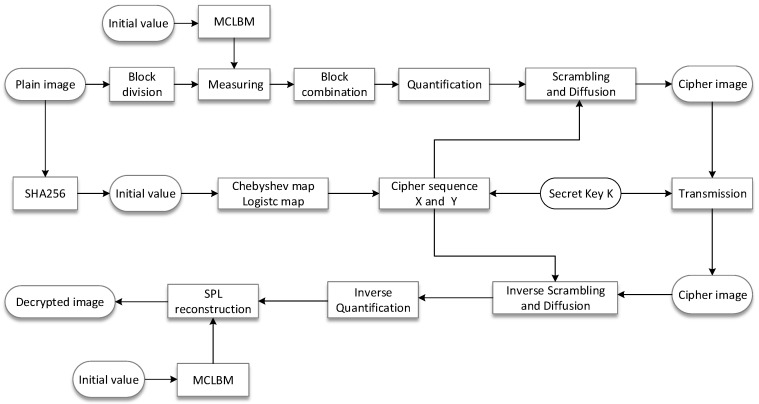
The proposed image encryption and decryption process.

The proposed compression/encryption algorithm consists of two main processes, BCS compression sampling and quantization encryption of the measured values, as shown in Figure 4. The specific steps are discussed in detail in the following sections.

### 3.1. Block Compression Sampling and Quantification Process

Assuming the BCS compression ratio is *CR*, the size of the plaintext image *P* is M×N, and it is divided into non-overlapping blocks of size B×B, which can be divided into $n_{B}=\frac{M\times N}{B\times B}$ blocks, and then these blocks are expanded into a one-dimensional vector in the scanning order from left to right and then from top to bottom, and noted as $S_{i},i=1,2,\cdots ,n_{B}$. The BCS compression sampling process is as follows:

1. Generate the MCLB sequence $\left\{z_{j},j=1,2,\cdots ,L\right\}$, from the steps in Section 2.2.2, and L=m×n, n=B×B, m=floor(CR×n).
2. Generate a measurement matrix $\Phi_{0}$ of size m×n by cyclically shifting $\left\{z_{j}\right\}$ by columns:
  (15) $$\Phi_{0}=\left(\begin{array}{cccc} z_{1} & z_{m+1} & \cdots & z_{mn-m+1}\\ z_{2} & z_{m+2} & \cdots & z_{mn-m+2}\\ \vdots & \vdots & \ddots & \vdots \\ z_{m} & z_{2m} & \cdots & z_{mn} \end{array}\right)$$
3. Express $\Phi_{0}$ as a row vector as $\Phi_{0}=\left(\alpha_{1}^{T},\alpha_{2}^{T},\cdots ,\alpha_{M}^{T}\right)$. Let $\beta_{1}=\alpha_{1}$ and use Equations (16) and (17) to normalize the row vectors.
  (16) $$\beta_{r}=\alpha_{r}-\sum_{i=1}^{r-1}\frac{\langle \beta_{i},\alpha_{r}\rangle }{\langle \beta_{i},\beta_{i}\rangle }\beta_{i}\;r=2,3,\cdots ,M$$
  (17) $$\eta_{j}=\frac{\beta_{j}}{\left\|\beta_{j}\right\|}j=1,2,\cdots ,M$$
  where 〈α,β〉 denotes the inner product of vector α and β, and ‖β‖ denotes the 2-norm of vector β. Finally, measurement matrix $\Phi_{i}=\left(\eta_{1}^{T},\eta_{2}^{T},\cdots ,\eta_{m}^{T}\right),\;i=1,2,\cdots ,n_{B}$ after orthogonal normalization is obtained.
4. Compress the plaintext image *P* after blocked and obtain the measurement value $C_{i}$ of each block.
  (18) $$C_{i}=\Phi_{i}S_{i}i=1,2,\cdots ,n_{B}$$

Finally, $C_{i}$ is combined in blocks to obtain the measurement value matrix *C*, at which point the measurement value matrix is

(19) $$C=\left(C_{1},C_{2},\cdots ,C_{n_{B}}\right)$$

At this time, the size of C is $m\times n_{B}$, and the data are obviously smaller than that of the original image. If the compression ratio is *CR* = 0.25, then the size of C is only 1/4 of the size of the plaintext image, which is convenient for the subsequent encryption processing.

The output matrix values after CS measurements are usually outside the grayscale range of [0, 255]. Therefore, it must be quantized before encryption. The quantization process is expressed as follows:

(20) $$P_{0}=\mathrm{floor}\left(\frac{255\times \left(Y-\min\right)}{\max-\min}\right)$$

where floor(x) represents the maximum integer value that is not greater than x and min and max are the minimum and maximum elements of the measurement matrix Y, respectively.

### 3.2. Cipher Sequence Generation Algorithm

In order to make the proposed encryption algorithm resistant to known plaintext and selected plaintext attacks, this paper uses the SHA-256 value of the plaintext image to calculate the parameters and initial values of the variable parameter chaotic mapping, so that the parameters and initial values are closely related to the plaintext image. The specific steps are as follows.

The 256-bit hash of the plaintext image is first obtained, written in 64-bit hexadecimal form, and then each bit is converted to the corresponding decimal number, which is used as the key K and is denoted as:

(21) $$K=k_{1},k_{2},\cdots ,k_{64}$$

where $k_{i}\in \left(0,16\right),i=1,2,\cdots ,64$. The encryption algorithm requires the use of Chebyshev chaotic map and the logistic chaotic map to generate two cipher sequences, so generate their initial values $x_{0}^{\prime }$, $\omega^{\prime }$, $y_{0}^{\prime }$, and $\lambda^{\prime }$ according to the key K, generated by the following equation.

(22) $$\{\begin{array}{l} x_{0}^{\prime }=-1\times \frac{1}{256}\sum_{i=1}^{16}k_{i}+1\\ \omega^{\prime }=2\times \frac{1}{256}\sum_{i=17}^{32}k_{i}\\ y_{0}^{\prime }=-2\times \frac{1}{256}\sum_{i=33}^{48}k_{i}+1\\ \lambda^{\prime }=-0.6\times \frac{1}{256}\sum_{i=49}^{64}k_{i}+2 \end{array}$$

Then the initial values $x_{0}^{\prime }$, $\omega^{\prime }$, $y_{0}^{\prime }$, and $\lambda^{\prime }$ generated by SHA-256 are substituted into Equations (4) and (6) to generate a chaotic sequence $S=\left\{s_{1},s_{2},\cdots ,s_{mn_{B}}\right\}$ and a chaotic sequence $Z=\left\{z_{1},z_{2},\cdots ,z_{{2mn}_{B}}\right\}$, respectively, after which the elemental values of the chaotic sequences S and Z are quantized using the following equation:

(23) $$\{\begin{array}{l} X=\mathrm{mod}\left(floor\left(\left(S+100\right)\times 10^{10}\right),m\times n_{B}\right)\\ Y=\mathrm{mod}\left(floor\left(Z\times 2^{16}\right),256\right) \end{array}$$

Sequence X is used for scrambling and Y for diffusion.

### 3.3. Image Encryption Algorithm

The encryption process begins with a scrambling of the matrix of measurement values, followed by a diffusion operation, as follows.

1. First, keep only one of the recurring pseudo-random numbers in the pseudo-random sequence X (i.e., the first occurrence), then add the values in $\left\{1,2,\cdots ,mn_{B}\right\}$ that do not appear in X to the end of X in descending order, and note $X_{1}$. $X_{1}$ is then used to displace C without repetition. C is then expanded column-wise into a one-dimensional vector, denoted $C_{1}$. Finally, $C_{1}\left(X_{1}\left(i\right)\right)$ is swapped with $C_{1}\left(X_{1}\left(mn_{B}-i+1\right)\right)$ to give the disordered matrix $C_{2}$.
2. Select the first $mn_{B}$ values of the sequence Y to form the sequence $Y_{1}$. $C_{3}$ is obtained by forward diffusion of $C_{2}$ using $Y_{1}$ as follows.
  (24) $$\{\begin{array}{lll} C_{3}\left(1\right)=C_{3}\left(0\right)\times Y_{1}\left(1\right)\times C_{2}\left(1\right) & & \\ C_{3}\left(i\right)=C_{3}\left(i-1\right)\times Y_{1}\left(i\right)\times C_{2}\left(i\right) & & i=2,3,\cdots ,mn_{B} \end{array}$$
  where “×” represents the multiplication operation of GF(257) domain and $C_{3}\left(0\right)=0$.
3. Select the values from $mn_{B}+1$ to $2mn_{B}$ of the sequence Y to form the sequence $Y_{2}$. Use $Y_{2}$ to perform backward diffusion on $C_{3}$ to obtain $C_{4}$, as follows.
  (25) $$\{\begin{array}{lll} C_{4}\left(mn_{B}\right)=C_{4}\left(0\right)\times Y_{2}\left(mn_{B}\right)\times C_{3}\left(mn_{B}\right) & & \\ C_{4}\left(i\right)=C_{4}\left(i+1\right)\times Y_{2}\left(i\right)\times C_{3}\left(i\right) & & i=mn_{B}-1,mn_{B}-2,\cdots ,1 \end{array}$$
  where “×” represents the multiplication operation of GF(257) domain and $C_{4}\left(0\right)=0$.

Finally, $C_{4}$ is rearranged into a matrix of $m\times n_{B}$ by columns, and after the above operation, the ciphertext image $C_{5}$ is obtained.

### 3.4. Image Decryption and Reconstruction Process

The decompression and decryption process is the opposite of the compression and encryption process. The steps are summarized as follows:

1. Use the key K to generate the chaotic mapping initial values $x_{0}^{\prime }$, $\omega^{\prime }$, $y_{0}^{\prime }$, and $\lambda^{\prime }$, and generate X and Y by Equation (23).
2. The received ciphertext image $C_{5}$ is sequentially subjected to backward diffusion, forward diffusion, and scrambling algorithms to obtain the quantized measurement value matrix $P_{0}$.
3. The measurement matrix C is recovered by inverse quantization of the quantized measurement matrix $P_{0}$ by the following equation:
  (26) $$C=\frac{P_{0}\times \left(\max-\min\right)}{255}+\min$$
4. The parameters ω, λ and the initial value $x_{0},\;y_{0}$ of the MCLB transmitted by the sender are used to iterate and generate the MCLBM, that is, the measurement matrix $\Phi_{i}$.
5. The DDWT-SPL algorithm is used to reconstruct the recovered measurement matrix C to obtain a reconstructed original image P of size M×N.

## 4. Simulation and Performance Analysis

### 4.1. Image Encryption Algorithm

To verify the effectiveness of the proposed scheme, simulation results and performance analyses are given in this section. The experiments were performed on a computer with 2.50 GHz CPU and 8 GB RAM using MATLAB R2017a. Different grayscale images (size 512 × 512) are selected for simulation tests, such as “Lenna”, “Peppers”, “Cell”, and “X-ray”. The initial value of MCLBM was set to ω=2, λ=4, $x_{0}=0.169877$, $y_{0}=0.2555$. The compression ratio of the compression measurement was *CR* = 0.25, and the size of the plain image blocks were all 32 × 32. In the decryption stage, DDWT-SPL was used as the reconstruction algorithm.

**Figure 5:**
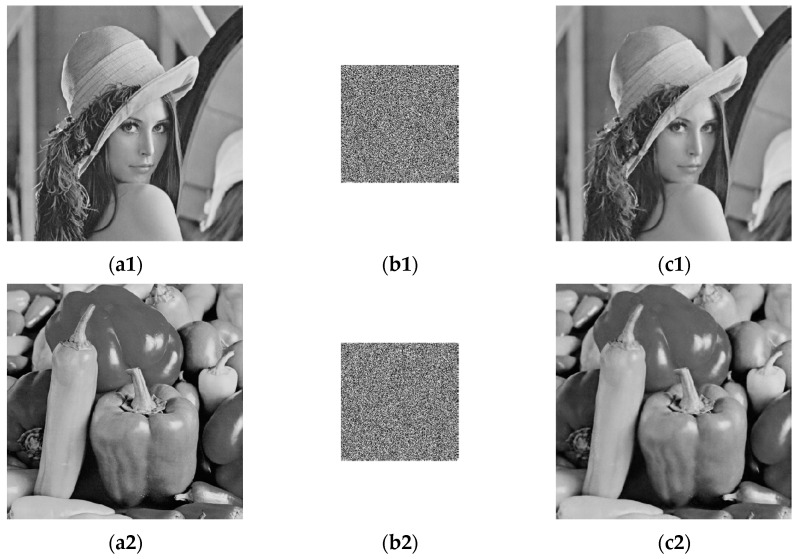

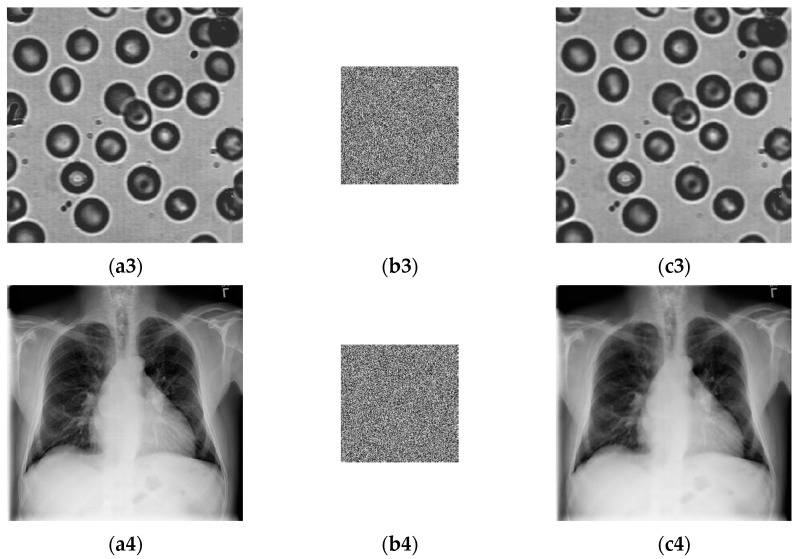
Simulation results. (**a1**–**a4**) Plaintext images; (**b1**–**b4**) ciphertext images; (**c1**–**c4**) decrypted images.

The simulation results are shown in Figure 5. The first to third columns represent the plaintext image, the encrypted image, and the decrypted reconstructed image, respectively. From the final encryption results, no meaningful information can be obtained from the ciphertext image in the third column. The compression and reconstruction results show that the size of the ciphertext image is only the size of the original image, which greatly reduces the image file size, storage space, and transmission bandwidth required for transmission. In addition, as shown in the third column, the decrypted reconstructed image is visually very similar to the corresponding plaintext image.

### 4.2. Impact of Important Parameters on Encryption and Decryption Performance

In this scheme, two important parameters, block size and compression ratio, both affect the encryption and decryption results. Next, we will evaluate their effects on the simulation results for better performance.

Firstly, the influence of block size on the reconstructed image PSNR is discussed when the compression ratio is the same. The “Lenna” image of 512 × 512 size was used for compressed encryption as well as decrypted reconstruction, with the compression ratio set to 0.25. Table 2 lists the PSNR values and the consumption time for different block sizes. It can be seen from Table 2 that when the compression ratio is the same, the PSNR value and the time consumed increase as the block size increases, but the PSNR value increases insignificantly and the time consumed increases too much, which has a side effect instead, so a high PSNR value can be achieved by choosing a block size of 32 × 32.

**Figure 6:**
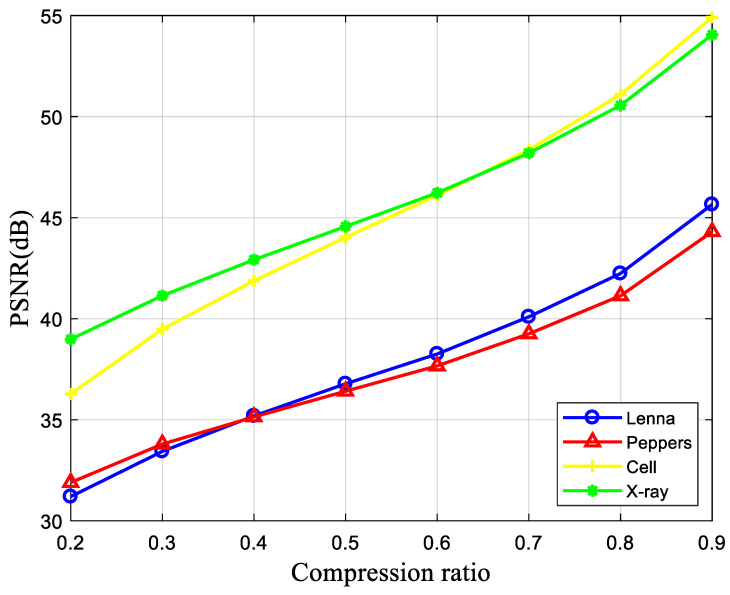
PSNR of reconstructed images with different compression ratios.

Then, we discuss the PSNR of the reconstructed image with a block size of 32 × 32 and different image compression–encryption and decryption at different compression ratios, as shown in Figure 6. It can be seen that the PSNR values of the different reconstructed images can still reach more than 30 dB at a compression ratio of 0.2, which indicates that the compression–encryption scheme proposed in this paper can achieve a high PSNR value at a low compression ratio.

Finally, the compression–aware compression ratio in the algorithm of this paper is set to 0.25 and the block size is 32 × 32. Table 3 compares the scheme of this paper with other compression–encryption schemes, and it can be seen that the algorithm proposed in this paper has higher PSNR values than other algorithms under the same compression ratio, which indicates that it has good compression reconstruction performance.

## 5. Statistical Analysis

### 5.1. Key Space

For a secure image encryption algorithm, the key space is at least $2^{100}$ to resist brute force attacks. In this scheme, the key consists of two parts: (1) the parameters ω, λ and the initial value $x_{0},\;y_{0}$ of the MCLB; and (2) the SHA-256 hash function of the plaintext image. According to the IEEE floating point standard, the precision of the double precision values is approximated as $10^{-15}$, SHA-256 key space can be up to $2^{128}$. Therefore, the key space of the proposed scheme in this paper is $10^{15}\times 10^{15}\times 10^{15}\times 10^{15}\times 2^{128}\approx 2^{300}$, greater than $2^{100}$. In other words, the key space of the algorithm meets the security criteria and can resist exhaustive attacks. Table 4 compares the key space of this paper with that of other compression–encryption schemes, and the results in Table 4 show that the proposed image encryption algorithm is secure enough to resist brute-force attacks.

### 5.2. Key Sensitivity

Key sensitivity analysis aims to analyze the difference between two ciphertext images obtained by encrypting the same plaintext image when there is a small change in the key. If the algorithm is sensitive to the key, it means that if the input decryption key is slightly different from the correct one when decrypting, no valuable information about the plain image can be obtained from the decrypted result. This section uses the plain image “Lena” as an example to test the key sensitivity of the algorithm. The sensitivity of the chaotic map to the initial conditions determines the sensitivity of the encryption system to the key. Therefore, the 256-bit hash value of the plaintext is changed from K to K_0_, K_1_, and K_2_ with bit modifications, which are shown below.

K = eb1197ae40dd90f096474ebf6a25494802027aa0a8b8f0f81c9d9a2e623c0b0a

K_0_ = fb1197ae40dd90f096474ebf6a25494802027aa0a8b8f0f81c9d9a2e623c0b0a

K_1_ = eb1197ae40dd90f006474ebf6a25494802027aa0a8b8f0f81c9d9a2e623c0b0a

K_2_ = eb1197ae40dd90f006474ebf6a25494802027aa0a8b8f0f81c9e9a2e623c0b0a

**Figure 7:**
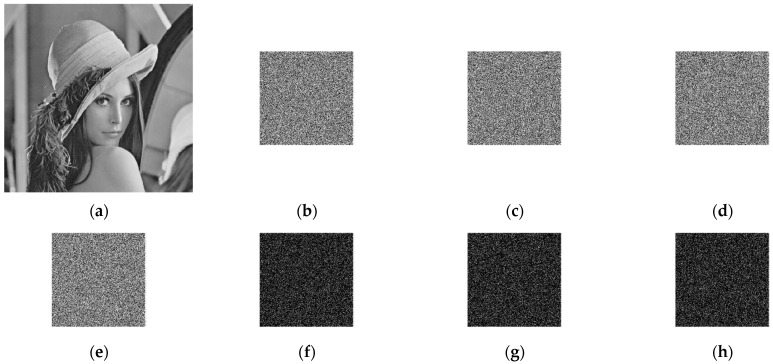

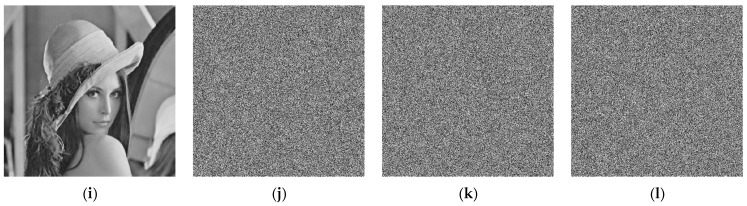
Results of wrong key encryption and decryption. (**a**) The plaintext image; (**b**–**e**) encrypted plaintext images using K, K_0_, K_1_, and K_2_, respectively; (**f**) Difference image of encrypted image ciphertext using K and K_0_; (**g**) Difference image of encrypted image ciphertext using K and K_1_; (**h**) Difference image of encrypted image ciphertext using K and K_2_; (**i**) Decrypt the (**b**) ciphertext image using K; (**j**–**l**) Decrypt the (b) ciphertext image using K_0_, K_1_, and K_2_, respectively.

The “Lena” image is encrypted with K to K_0_, K_1_, and K_2_ to obtain different cipher images, as shown in Figure 7b–e. Figure 7f–g shows the difference between two ciphertexts; as seen from Figure 7f–g, the “difference image” of two ciphertexts obtained by encrypting the same plaintext image shows a noise style, intuitively, it reflects that the two ciphertexts differ significantly; that is, the image encryption system has key sensitivity. Figure 7j–l shows the images decrypted by different “wrong” keys; it can be seen that the information of the original image cannot be obtained in the decrypted images, which indicates that the image decryption system is key sensitive.

To quantify the differences between ciphertext images obtained from the same plaintext image, the pixel change range (NPCR) and the uniform average change intensity (UACI) were calculated using the correct and incorrect keys for encrypting the images, and the results are shown in Table 5. NPCR and UACI are calculated as follows.

(27) $$\left\{ \begin{array}{l} \mathrm{NPCR}=\frac{1}{M\times N}\sum_{i=1}^{M}\sum_{j=1}^{N}D(i,j)\\ \mathrm{UACR}=\frac{1}{M\times N}\sum_{i=1}^{M}\sum_{j=1}^{N}\frac{\left|C1(i,j)-C2(i,j)\right|}{255} \end{array}\right.$$

where, *D*(*i*,*j*) is the Heaviside function, *D*(*i*,*j*) = 0 when *C*1(*i*,*j*) = *C*2(*i*,*j*), and *D*(*i*,*j*) = 1 when *C*1(*i*,*j*) ≠ *C*2(*i*,*j*). The theoretical expectation of NPCR is 996.6034% and the theoretical expectation of UACI is 33.4635%.

Table 5 shows that both NPCR and UACI are close to the theoretical values and more than 99.6% of the pixels are modified, which means that the encryption process is highly sensitive to the key.

Table 6 lists the NPCR and UACI values in Figure 7i–l, where more than 99.5% of the pixels are changed when the key is slightly changed, which indicates that the decryption process is highly sensitive to the key.

### 5.3. Histogram

**Figure 8:**
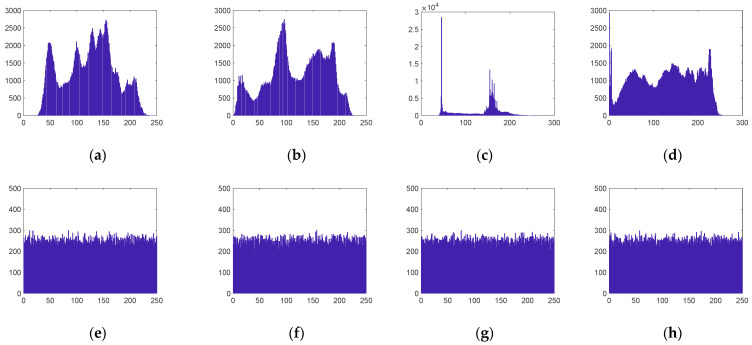
Histograms of the original and encrypted images. (**a**) Plaintext histogram of Lena; (**b**) plaintext histogram of Peppers; (**c**) plaintext histogram of Cell; (**d**) plaintext histogram of X-ray; (**e**) ciphertext histogram of Lena; (**f**) ciphertext histogram of Peppers; (**g**) ciphertext histogram of Cell; (**h**) ciphertext histograms of X-ray.

The histogram is a fundamental property of digital images. It reflects the distribution of pixel grayscale values in the image. The more uniform the histogram distribution of the ciphertext image, the better the encryption effect [9]. As can be seen from Figure 8, the pixel value distributions of different plaintext images have their own characteristics, and their ciphertext image histograms are all approximately uniformly distributed, so that the attacker cannot obtain any statistical information from the ciphertext image. Therefore, the image compression and encryption algorithm proposed in this paper is able to resist statistical analysis attacks.

### 5.4. Information Entropy Analysis

The information entropy reflects the uncertainty of the image information, and it is generally believed that the greater the entropy, the greater the uncertainty, and the greater the amount of information, the smaller the visible information. The formula for calculating information entropy is shown below:

(28) $$H=-\sum_{i=0}^{L}p\left(i\right)\log_{2}p\left(i\right)$$

where L is the gray level of the image and p(i) denotes the probability of occurrence of the gray value i.

For a grayscale random image with L = 256, the theoretical value of information entropy is 8. Table 7 presents the information entropy of different cipher images for different compression ratios. From Table 7, it can be seen that the information entropy of each cipher image is very close to the theoretical value, even under different compression ratios. Therefore, the compression–encryption scheme proposed in this paper can achieve both image compression and encryption, and the encrypted images have good randomness and sufficient security.

### 5.5. Correlation Analysis

The correlation coefficient of adjacent pixels can be used to evaluate the image encryption effectiveness. Generally, a relatively good digital image encryption scheme can make the correlation between adjacent pixels of the ciphertext image relatively low [58,59]. The closer the correlation coefficient of adjacent pixels to 0, the better the encryption effect. In this paper, for 10,000 pairs of pixels selected in the image, the correlation coefficient of adjacent pixels is calculated by the formula:

(29) $$\{\begin{array}{l} r_{uv}=\frac{\mathrm{cov}\left(u,v\right)}{\sqrt{D(u)}\sqrt{D(v)}}\\ \mathrm{cov}(u,v)=\frac{1}{N}\sum_{i=1}^{N}\left(x_{i}-E(u))(y_{i}-E(v)\right)\\ D(u)=\frac{1}{N}\sum_{i=1}^{N}{\left(u_{i}-E(u)\right)}^{2}\\ E(u)=\frac{1}{N}\sum_{i=1}^{N}u_{i} \end{array}$$

where N denotes any N pair of adjacent pixel points in the image, $(u_{i},v_{i}),i=1,2,\cdots ,N$ denotes the grayscale value of N pairs of adjacent pixel points, E(u) and E(v) are the averages of all u and v. In addition, D(u) is the variance, cov(u,v) is the covariance, and $r_{uv}$ is the correlation coefficient. Lenna, Peppers, Cell, and X-ray of size 512 × 512 were used as plaintext images in this experiment. To calculate the correlation coefficients, 10,000 pairs of pixels were randomly selected from the plaintext and ciphertext images in horizontal, vertical, and diagonal directions. The calculation results are shown in Table 8.

**Figure 9:**
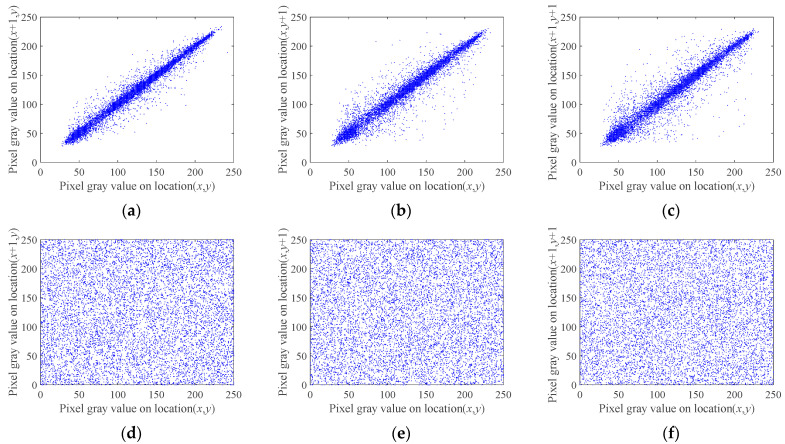
Distribution of adjacent pixels. (**a**) Plaintext horizontal adjacent pixels; (**b**) Plaintext vertical adjacent pixels; (**c**) Plaintext diagonal adjacent pixels; (**d**) Ciphertext horizontal adjacent pixels; (**e**) Ciphertext vertical adjacent pixels; (**f**) Ciphertext diagonal adjacent pixels.

As can be seen from Table 8, the correlation coefficients of neighboring pixels in the plain images in all three directions are greater than 0.9, indicating that the correlation between neighboring pixels is high. The correlation of adjacent pixels in the images encrypted with the algorithm proposed in this paper is close to 0. Using Lena as an example, 10,000 pairs of pixels were selected from the original and encrypted images in the horizontal, vertical and diagonal directions. Their adjacent pixel correlations in horizontal, vertical, and diagonal directions are plotted in Figure 9, which shows the pixel distribution before and after encryption of the Lena image; it can be seen that the pixel distribution follows a certain pattern, and the grayscale values from the ciphertext image are randomly distributed between 0 and 255.

### 5.6. Choose Plaintext Attack

According to Kerckhoffs’s principle, the security of an encryption system depends on the key and not on the encryption algorithm itself. The choose plaintext attack [10,54,60], implies that an attacker temporarily gains access to use the encryption machine, so that he can encrypt any plaintext and obtain the corresponding ciphertext to decrypt all (or part) of the plaintext and the key. The chosen plaintext attack is the strongest attack, and if a cryptosystem can resist this attack, then it must be able to resist other attacks. In this paper, the SHA-256 hash function of the original image was calculated as the initial value of the chaotic system. Therefore, when different plaintext images are encrypted, the corresponding encryption sequences change, so that a hacker cannot obtain useful information by encrypting some specific images. Therefore, the scheme can resist well to selective plaintext attacks.

### 5.7. Ciphertext Sensitivity

The purpose of ciphertext sensitivity analysis is to analyze the difference between the restored image and the original plaintext image after a small change in the ciphertext image by the decryption system. If the restored image is very different from the original plaintext image, the image decryption system is said to have strong ciphertext sensitivity; if the restored image is not very different from the original plaintext image, the image cryptosystem is said to have weak ciphertext sensitivity, and then this type of image cryptosystem is deficient in fighting against selected ciphertext attacks or known ciphertext attacks. Moreover, a small change in the ciphertext image is a small change in the value of one or more pixel points of a given ciphertext image.

In this paper, different plaintext images P were selected, and the corresponding ciphertext image $C_{1}$ was obtained by encrypting P using this encryption system. A pixel point was randomly selected from $C_{1}$ and the value of this pixel point was changed by the amount of 1, the changed image was noted as $C_{2}$, and the restored image $P_{1}$ was obtained by decrypting $C_{2}$. The NPCR and UACI values of P and $P_{1}$ were calculated and listed in Table 9.

From Table 9, we can see that the calculated results of both NPCR and UACI are close to their respective theoretical values, indicating that the encryption system has strong ciphertext sensitivity.

### 5.8. Time Complexity Analysis

The algorithm differs from the traditional compressive sensing algorithm in the use of block compressive sensing, and in the image recovery process, the DDWT-SPL algorithm is used. The encryption and decryption times of Lena and Peppers images of size 512 × 512, with different compression ratios (*CR*), were analyzed; the results are shown in Table 10.

Table 11 shows the comparison of the encryption time of the algorithm in this paper with other algorithms for the compression ratio *CR* = 0.25 (encrypt ’’Lena’’ image). It can be seen from Table 11 that the algorithm is faster than other BCS image encryption algorithms [61] when encrypting images of the same size. Compared to the image encryption algorithms based on the chaotic measurement matrix and SHA-256 [16], the image compression–encryption algorithm in this paper is slightly slower.

Next, the time complexity of the encryption scheme proposed in Section 3 was analyzed in detail, assuming that the size of the plaintext image was M×N, the size of the block compression–aware measurement matrix was m×n, and the compression ratio was CR. Section 3.1, involved generating the measurement value matrix, and the time complexity of generating the MCLB is Θ(2×m×n), the time complexity of generating the measurement matrix is Θ(m×n), and the time complexity of the final quantized measurement value matrix is Θ(CR×M×N). Section 3.2 focused on generating the cipher sequence, where the time complexity of generating the key value was Θ(M×N), the time complexity of generating two cipher sequences was Θ(2×CR×M×N). Section 3.3 involved the encryption of the measurement value matrix, where the time complexity of the permutation of the measurement values was Θ(CR×M×N), and then we diffused it, with a time complexity of Θ(2×CR×M×N). Then the total time complexity was Θ(2×M×N). Compared with the [55,61] encryption algorithms listed in Table 12, our scheme has a smaller time complexity.

## 6. Conclusions

A block compressive sensing image encryption algorithm based on the mixed chaotic Bernoulli measurement matrix is proposed. The core of this encryption algorithm is the combination of the mixed chaotic sequence measurement matrix and BCS, which is practical and can improve the reconstruction quality of images with low compression ratios under the premise of ensuring system security and easy hardware implementation. The compression performance of the mixed chaotic sequence measurement matrix was analyzed, and the performance analysis shows that the measurement matrix has better measurement reconstruction performance than the ordinary measurement matrix. In the encryption process, the SHA-256 function of the plaintext image was used to generate the initial values of the desired chaotic sequence, which improved the ability of the scheme to resist the selective plaintext attack. Finally, the results of the encryption scheme were analyzed and evaluated. First, the effect of reconstructing the image was analyzed; the method has better reconstruction capabilities at a low compression ratio compared with other methods, which could greatly reduce the amount of data to be processed. Secondly, the security of the encryption algorithm was analyzed. The encryption algorithm could resist various attacks from key space, statistical analysis, and information entropy. Finally, the proposed encryption scheme has good encryption effects and image compression capabilities.

## Figures and Tables

**Table 1:** Performance comparison of different measurement matrices at a compression ratio of 0.3.

Measurement Matrix	PSNR/dB	Time/s
GM	32.82	3.50
BM	32.25	3.70
LBM	32.45	3.21
CBM	32.57	3.56
MCLBM	33.82	3.18

**Table 2:** Comparison of PSNR and consumption time for different block sizes with the same compression ratio.

Block Size	PSNR/dB	Time/s
32 × 32	32.7265	8.955823
64 × 64	32.9089	12.1209232
128 × 128	33.1612	153.747594

**Table 3:** PSNR/dB of different algorithms.

Image	*CR*	Ours	Reference [55]	Reference [56]
Lena	0.25	32.7265	31.4240	\
Peppers		32.9463	30.6809	\
Lena	0.5	36.7805	33.2299	23.3608
Peppers		36.4142	32.1889	27.3366
Lena	0.75	41.0781	34.1313	34.7149
Peppers		40.1133	33.1721	35.8463

**Table 4:** Comparison of key space.

Algorithm	Ours	Reference [55]	Reference [57]	Reference [58]
Key space	2^300^	6.561 × 10^87^	10^80^	10^75^

**Table 5:** NPCR and UACI for cryptographic image using the correct key and different incorrect keys.

Secret Keys	K and K_0_	K and K_1_	K and K_2_
NPCR (%)	99.6223	99.6074	99.6013
UACI (%)	33.4190	33.4347	33.3847

**Table 6:** NPCR for decrypting images using the correct key and different incorrect keys.

Secret Keys	K and K_0_	K and K_1_	K and K_2_
NPCR (%)	99.5671	99.5725	99.6372

**Table 7:** Information entropy of different cipher.

*CR*	Lena	Peppers	Cell	X-ray
0.25	7.9972	7.9971	7.9975	7.9975
0.5	7.9986	7.9988	7.9986	7.9983
0.75	7.9990	7.9990	7.9991	7.9989

**Table 8:** Correlation coefficients of neighboring pixels in different images.

Direction	Lenna		Peppers		Cell		X-ray	
	Plaintext	Ciphertext	Plaintext	Ciphertext	Plaintext	Ciphertext	Plaintext	Ciphertext
Horizontal	0.9845	−0.0032	0.9751	0.0075	0.9893	0.0163	0.9985	0.0410
Vertical	0.9746	0.0123	0.9763	0.0129	0.9904	−0.0059	0.9976	−0.0148
Diagonal	0.9703	−0.0071	0.9649	−0.0051	0.9792	0.0215	0.9964	−0.0082

**Table 9:** Ciphertext sensitivity analysis results.

Images	NPCR (%)	UACI (%)
Lena	99.6246	33.5234
Peppers	99.6109	33.4788
Cell	99.6265	33.4929
X-ray	99.6147	33.4934

**Table 10:** Image encryption and decryption time(s) at different compression ratios.

Images	Lena		Peppers	
Process	Encryption	Decryption	Encryption	Decryption
*CR* = 0.25	1.007266	7.948557	0.903141	7.736676
*CR* = 0.5	1.344250	5.933346	1.190280	6.051189
*CR* = 0.75	1.640416	3.167031	1.956602	3.195703

**Table 11:** Image encryption and decryption speed of different schemes at *CR* = 0.25.

Algorithm	Time/s	
Process	Encryption	Decryption
Ours	1.007266	7.948557
Reference [61]	5.380744	12.175169
Reference [16]	0.4115	3.5099

**Table 12:** Comparison results of time complexity.

Algorithm	Time Complexity
Ours	Θ(2×M×N)
Reference [55]	Θ(5×M×N)
Reference [61]	Θ(5×M×N)

## Data Availability

Data is contained within the article.
